# Case Report: Laparoscopic appendectomy in a patient with congenital visceral heterotaxy and severe adhesions

**DOI:** 10.3389/fsurg.2025.1702385

**Published:** 2025-12-01

**Authors:** Dake Liu, Xinying Zhao, Lin Li, Weibo Liu

**Affiliations:** Shijiazhuang People’s Hospital, Shijiazhuang, China

**Keywords:** heterotaxy syndrome, laparoscopic appendectomy, surgical adhesions, cesarean section history, appendicitis

## Abstract

**Background:**

Congenital visceral heterotaxy (CVH) is a rare congenital anomaly characterized by the abnormal arrangement (malposition) of thoracic and abdominal organs. This condition can significantly complicate surgical planning and intraoperative management, particularly when a history of prior abdominal surgery is present and laparoscopic intervention is required. The limited literature on managing such cases underscores the necessity for meticulous preoperative planning and intraoperative adaptability.

**Case summary:**

A 42-year-old Chinese woman with congenital visceral heterotaxy and a history of cesarean section 11 years prior was admitted with acute suppurative appendicitis. Preoperative imaging, including ultrasound and CT, confirmed the diagnosis and revealed severe intra-abdominal adhesions. The patient underwent a laparoscopic appendectomy under general anesthesia. During the procedure, adhesions—likely stemming from the prior cesarean section—significantly impeded visualization and access to the appendix. Despite these challenges, the appendectomy was completed successfully without complications. The patient's postoperative recovery was uneventful, and she was discharged on the third postoperative day.

**Conclusion:**

This report highlights the successful management of a complex case involving a patient with both congenital visceral heterotaxy and severe intra-abdominal adhesions. It demonstrates that laparoscopic surgery is a feasible and safe option in such challenging scenarios, emphasizing the critical importance of meticulous preoperative planning, intraoperative adaptability, and a patient-tailored surgical approach. This case contributes to the sparse literature on laparoscopic surgery in patients with CVH and a history of abdominal surgery, offering valuable insights for surgeons facing similar complex presentations.

## 1 Introduction

Congenital visceral heterotaxy (CVH) is a rare congenital disorder characterized by the abnormal positioning of the thoracic and abdominal organs, which deviate from the typical anatomical arrangement (1). This condition is primarily classified into three distinct types: situs inversus, situs ambiguus, and partial situs inversus (2). CVH occurs in approximately 1 in 10,000 to 1 in 50,000 live births, making it a rare but clinically significant condition (3). Patients with CVH often present with associated congenital anomalies, such as cardiac defects and gastrointestinal malformations, which further complicate their clinical management. The diagnosis of CVH is typically confirmed through imaging studies, including echocardiography, CT, and MRI (4). Although situs inversus is the most common form, situs ambiguus and partial situs inversus are associated with a higher risk of organ dysfunction and pose greater surgical challenges.

## 2 Case report

### 2.1 Timeline with relevant data from the episode of treatment

**Figure 1:**
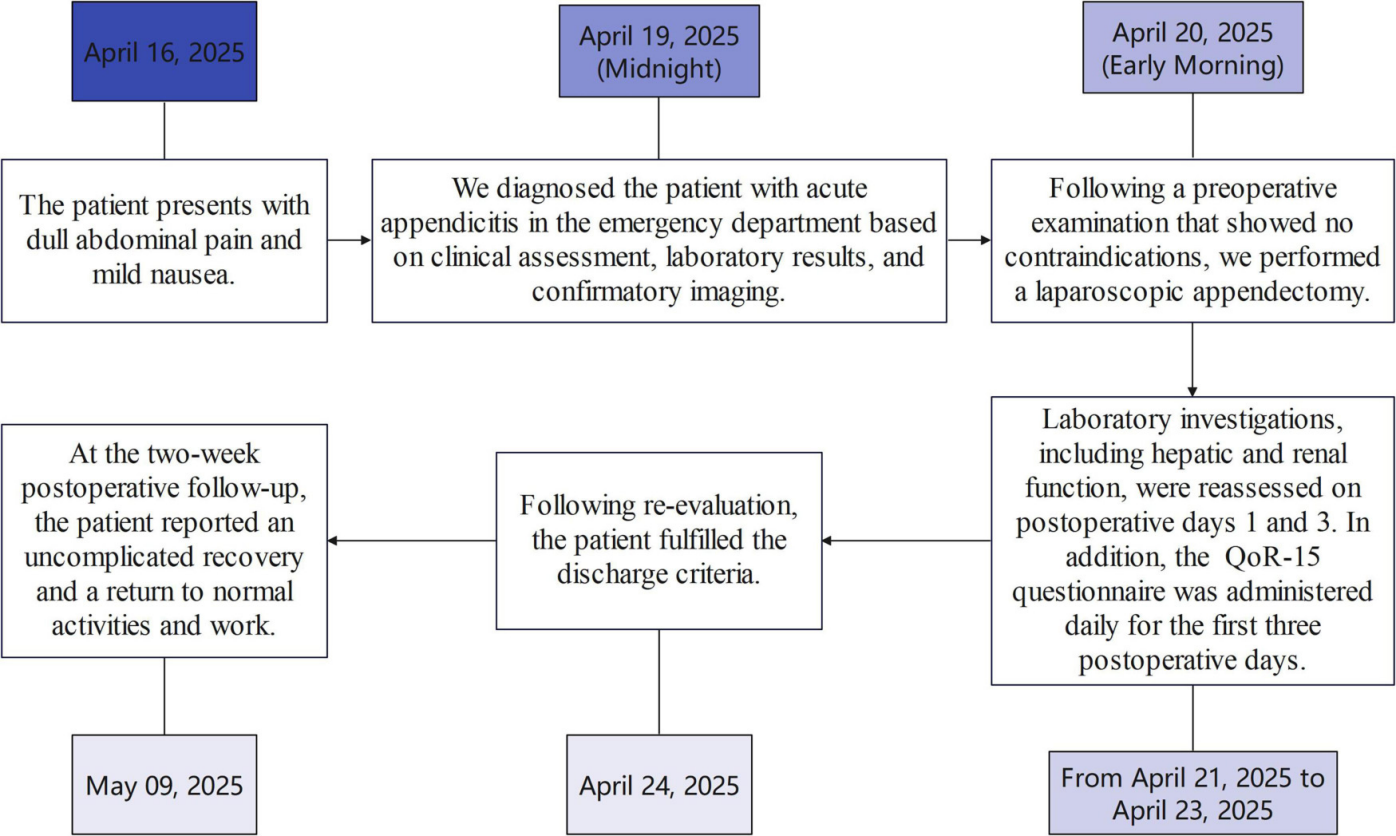
Timeline of treatment.

See Figure 1 for reference.

### 2.2 Patient history

A 42-year-old Chinese woman presented to the hospital with a 3-day history of abdominal pain that had migrated to the left lower quadrant. The pain began suddenly as a persistent, dull ache in the upper abdomen without an obvious precipitating factor and was accompanied by mild nausea. Approximately 24 h before admission, the pain shifted and became localized to the left lower quadrant, prompting her presentation. She reported no vomiting, diarrhea, or recent changes in bowel habits. Her past medical history was significant for CVH, diagnosed in childhood, and a cesarean section performed 11 years earlier. She denied any other abdominal surgeries or chronic medical conditions. The patient reported a known allergy to cephalosporins and was not on any long-term medications. Her family history was unremarkable for predisposing factors, congenital anomalies, or gastrointestinal diseases.

### 2.3 Physical examination

On admission, the patient's vital signs were as follows: a low-grade fever of 37.5 °C, a heart rate of 90 beats per minute, and a blood pressure of 119/91 mmHg. Abdominal examination revealed localized tenderness and rebound tenderness in the left lower quadrant, without muscle guarding. Bowel sounds were normoactive, and no masses were palpated. The remainder of the physical examination was unremarkable.

### 2.4 Imaging studies

**Figure 2:**
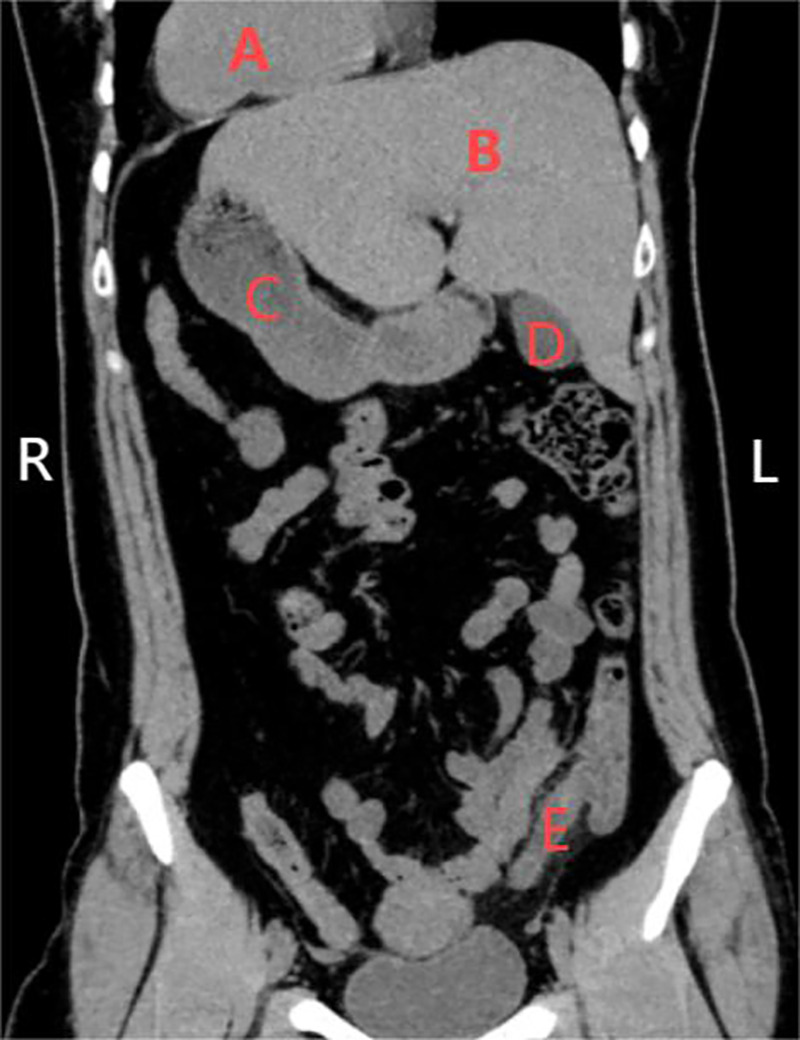
CT scan results upon admission. In the figure: **(A)** is the heart, **(B)** is the liver, **(C)** is the stomach, **(D)** is the gallbladder, and **(E)** is the appendix, which is inflamed.

Following admission, non-contrast-enhanced chest and abdominal-pelvic CT scans were performed. The imaging studies revealed an enlarged appendix with irregular margins and a hyperdense linear area measuring approximately 8 mm in diameter at the base of the appendix, consistent with acute appendicitis and the presence of an appendiceal fecolith. Additionally, the CT scan confirmed the presence of visceral heterotaxy involving both the thoracic and abdominal cavities, thereby confirming the diagnosis of CVH. The specific findings are detailed in Figure 2.

### 2.5 Laboratory biochemical testing

Upon admission, the patient underwent laboratory biochemical tests. Blood tests revealed a white blood cell (WBC) count of 9.40 × 10^9^/L (normal range: 3.50–9.50 × 10^9^/L) and a neutrophil count of 7.17 × 10^9^/L (normal range: 1.8–6.3 × 10^9^/L), suggesting a mild infection. The red blood cell (RBC) count was 4.44 × 10^12^/L (normal range: 4.3–5.8 × 10^12^/L), hemoglobin was 125 g/L (normal range: 130–175 g/L), and platelet count was 234 × 10^9^/L (normal range: 125–350 × 10^9^/L), all within normal ranges. C-reactive protein (CRP) was significantly elevated at 19.10 mg/L (normal range: <6.0 mg/L), indicating an acute inflammatory response. Liver and kidney function tests were within normal limits. Coagulation parameters and blood and urine amylase levels were normal, ruling out acute pancreatitis. These findings supported a diagnosis of acute appendicitis, with no evidence of other systemic diseases or complications.

### 2.6 Surgical procedure

**Figure 3:**
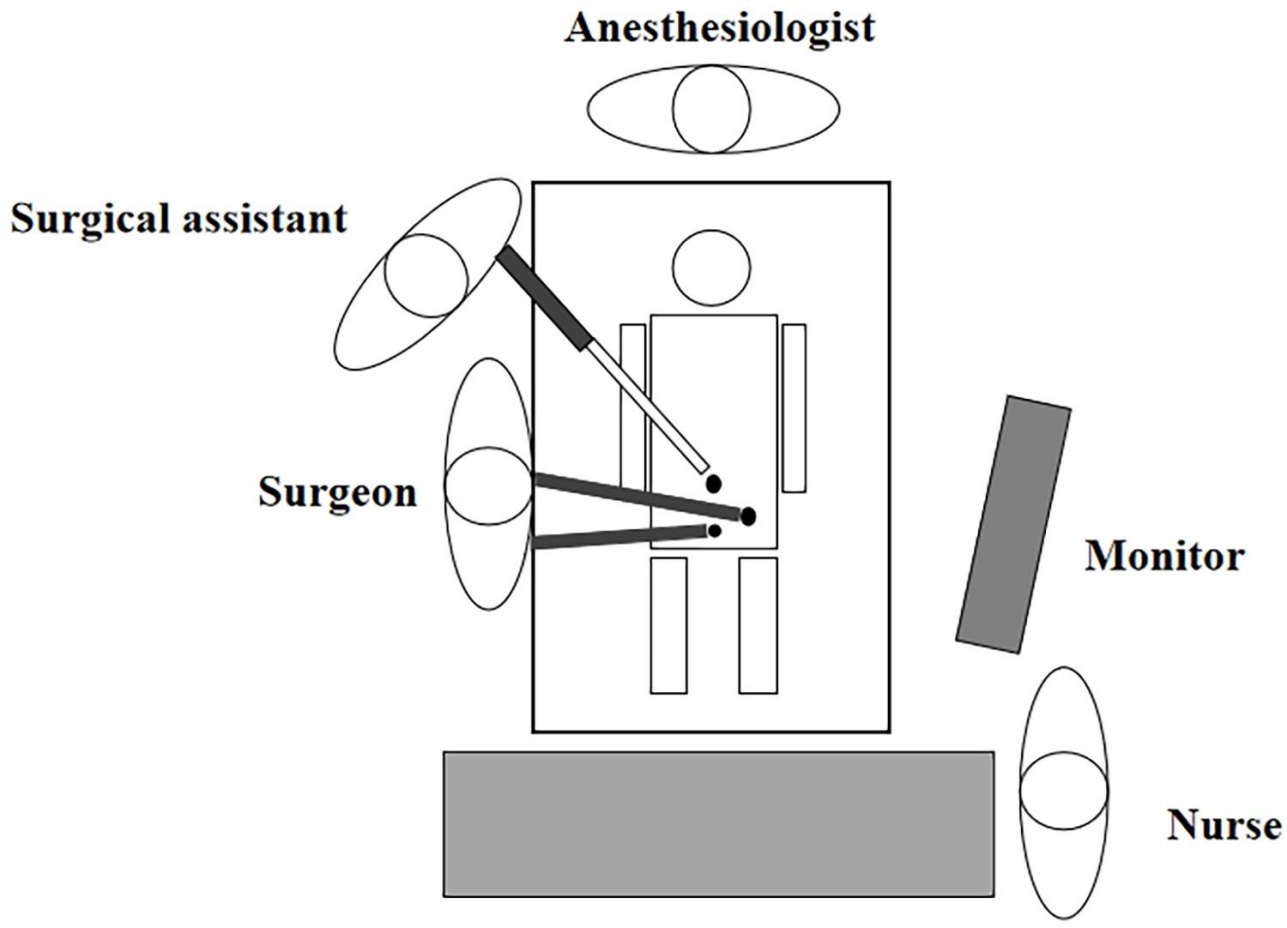
Location diagram of medical and nursing personnel during surgery.

An emergency laparoscopic appendectomy was performed. Due to the CVH, the intra-abdominal anatomy was distorted, with the appendix situated in the left lower quadrant. This required the surgical team to adjust their surgical technique during the procedure. The specific intra-abdominal anatomy and surgical approach are illustrated in Figure 3 (5).

**Figure 4:**
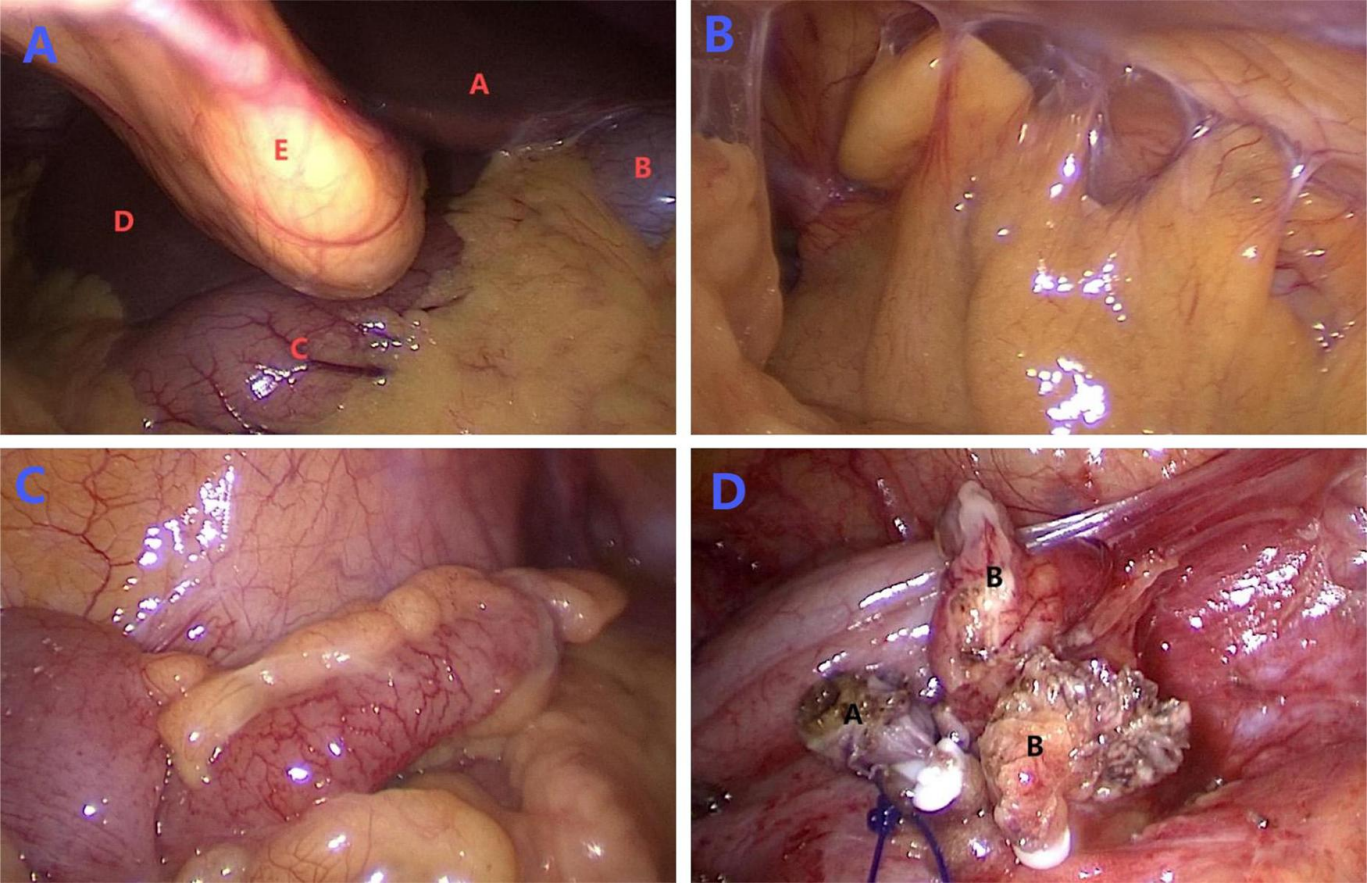
Intraoperative laparoscopic findings. Figure **(A)** shows the initial view of the abdominal cavity during surgery. Red letters identify the anatomical structures as follows: **(A)** corresponds to the left lobe of the liver, **(B)** marks the gallbladder, **(C)** identifies the stomach, **(D)** corresponds to the right lobe of the liver, and **(E)** marks the falciform ligament. Figure **(B)** shows the adhesion situation in the abdominal cavity. Figure **(C)** shows the complete view of inflamed and swollen appendix visible under laparoscopy. Figure **(D)** illustrates the post-appendectomy findings, with black letter **(A)** indicating the appendiceal remnant and black letter **(B)** marking the separated mesentery.

During the procedure, significant intra-abdominal adhesions, likely attributable to the prior cesarean section, were encountered. These adhesions compromised the visualization and obscured the identification of the appendix. To address this, adhesiolysis was meticulously performed using electrocautery and sharp dissection, which significantly improved exposure. The appendix was found to be inflamed, distended, and surrounded by purulent exudate. The operation was a standard laparoscopic appendectomy, involving ligation at the base of the appendix and its resection. The operation lasted 40 min with minimal blood loss of 5 mL. The specific situation during the operation is shown in Figure 4.

The patient's postoperative care adhered to an established Enhanced Recovery After Surgery (ERAS) protocol to ensure safety and promote rapid recovery (6). Vital signs were continuously monitored until clinical stability was achieved. Management included intravenous fluid and electrolyte replacement, provision of adequate analgesia, and encouragement of early ambulation. Due to the patient's cephalosporin allergy, antimicrobial prophylaxis was maintained with levofloxacin (200 mL twice daily). Postoperative recovery was assessed daily using the QoR-15 evaluation scale by dedicated nursing staff, in accordance with standard criteria (7).

On the first postoperative day, laboratory tests revealed a elevated WBC count of 10.43 × 10^9^/L (normal range: 3.50–9.50 × 10^9^/L) and neutrophil count of 9.19 × 10^9^/L (normal range: 1.8–6.3 × 10^9^/L), with a serum CRP level of 62.20 mg/L (normal range: <6.0 mg/L). No other significant abnormalities were noted. The patient's QoR-15 score was 108. She reported an acceptable mental and sleep state but had not yet passed flatus. No fever, nausea, or vomiting was recorded. The abdominal drain output was 5 mL of serosanguinous fluid, consistent with expected postoperative drainage.

On the second postoperative day, the patient's recovery progressed well. She passed flatus and reported only mild nausea without vomiting. A liquid diet was initiated and tolerated. Drain output increased to 20 mL of serosanguinous fluid, which remained within normal expectations.

On the third postoperative day, the patient was in good spirits, experienced no discomfort, and blood tests were reassessed. The WBC count was 5.63 × 10^9^/L (normal range: 3.50–9.50 × 10^9^/L), neutrophils were 3.16 × 10^9^/L (normal range: 1.8–6.3 × 10^9^/L), and serum CRP was 22.30 mg/L (normal range: <6.0 mg/L), with no other significant abnormalities. The QoR-15 quality of life scale was used to assess recovery, achieving a score of 125 points, representing a 17-point improvement from the first postoperative day. The abdominal drainage tube was removed on the same day.

The patient was discharged on the morning of the fourth postoperative day. The postoperative pathology report confirmed a diagnosis of acute suppurative appendicitis.

### 2.7 Outcome

In a 2-week follow-up, the patient reported complete resolution of symptoms with no evidence of surgical complications. She has fully recovered and resumed normal activities.

## 3 Discussion

CVH is a rare congenital anomaly characterized by the complete or partial reversal of thoracic and abdominal organ positions. This condition poses unique challenges for preoperative preparation and surgical planning, particularly when combined with a history of prior surgery, such as a cesarean section. The abnormal positioning of organs requires surgeons to possess exceptional spatial awareness and anatomical knowledge, especially when performing minimally invasive procedures like laparoscopic surgery. Advanced preoperative imaging techniques, such as CT scans, are crucial for mapping the patient's specific anatomy and guiding surgical planning. While CVH patients often present with additional systemic abnormalities, such as congenital heart defects (8), which can elevate surgical risks, the absence of such comorbidities in this case provided favorable conditions for a successful procedure. This case underscores the critical importance of meticulous preoperative planning, advanced imaging techniques, and specialized surgical skills when managing rare conditions like CVH.

The patient's history of cesarean section 11 years prior had resulted in significant intra-abdominal adhesions, which increased surgical complexity by elevating the risks of bowel or vascular injury and potentially prolonging the operation. These factors collectively heightened the possibility of postoperative complications. To address this challenge, the surgical team implemented the following key strategies to ensure a successful outcome: (1) Meticulous adhesiolysis: Adhesions were carefully divided using electrosurgical instruments, with continuous attention to preserving the integrity of surrounding organs and vasculature, thereby minimizing the risk of inadvertent injury and bleeding. (2) Enhanced visualization: The laparoscopic viewing angles and patient positioning were dynamically adjusted to optimize exposure of the operative field, facilitating safer and more effective adhesion management. (3) Team collaboration: Seamless collaboration among surgical team members ensured efficient adhesion separation, improving both safety and procedural efficiency. Through the strict application of these strategies, the severe adhesions were successfully managed, allowing for the completion of an uncomplicated laparoscopic appendectomy.

Laparoscopic surgery demonstrated distinct advantages over traditional open surgery in this complex case. The laparoscopic approach provides well-established benefits, including minimal invasiveness, accelerated recovery, and reduced postoperative pain (9). Its successful application here confirms its feasibility and safety in managing complex presentations like this one, thereby optimizing the patient's postoperative experience. However, laparoscopic surgery in such contexts has inherent challenges. Anatomical anomalies such as visceral heterotaxy, combined with adhesions, can prolong operative time and demand a higher level of surgical expertise. Consequently, comprehensive preoperative planning and the ability to adapt intraoperatively are crucial for ensuring a successful outcome.

A review of the literature indicates that while this case shares features with previously reported ones, it is distinguished by its unique surgical management and postoperative recovery course. Existing studies rightly emphasize the heightened technical difficulties of laparoscopic surgery in CVH patients (10). This case, consistent with the findings of Jin et al. (11), confirms that such challenges can be overcome with a tailored approach, exemplified by customized trocar placement. Furthermore, our experience extends the current understanding: whereas prior research, such as that by Sheik-Ali et al. (12), has focused on navigating congenital malformations, our case underscores that the strategic management of acquired conditions—particularly severe adhesions from prior surgery—is equally critical for success. Thus, this report reinforces that meticulous and adaptive preoperative planning is indispensable for achieving favorable outcomes in the laparoscopic management of complex CVH cases.

The successful management of this case offers valuable insights for similar clinical presentations. In contrast to standard appendectomies, our experience underscores several critical adaptations: comprehensive preoperative imaging was indispensable for delineating not only the congenital CVH anatomy but also, and more critically, the extent of post-surgical adhesions. Furthermore, the strategic and meticulous laparoscopic lysis of these severe adhesions constituted a pivotal procedural difference from managing uncomplicated heterotaxy and was a major factor contributing to the successful outcome. This case confirms that laparoscopic surgery is a viable and effective option in such complex scenarios, facilitating an optimal outcome and a rapid recovery while avoiding the potential complications associated with such high-risk anatomy. The primary educational value of this report lies in demonstrating a structured and successful protocol for safely applying minimally invasive surgery to patients facing the dual challenge of CVH and significant acquired adhesions.

## 4 Patient perspective

As a patient with congenital visceral heterotaxy—a rare anatomical condition—and a history of cesarean section, I was deeply anxious upon being diagnosed with acute appendicitis. I feared the complexity of the surgery and the associated risks. However, the medical team thoroughly addressed my concerns with detailed preoperative evaluations and a well-structured surgical plan. The laparoscopic procedure was a success. Despite the challenges presented by my unique anatomy and prior surgery, the operation proceeded smoothly. My recovery was rapid and free of complications, allowing me to return to my normal life and work shortly after discharge.

## Data Availability

The raw data supporting the conclusions of this article will be made available by the authors, without undue reservation.
